# Utilizing Feline Lentiviral Infection to Establish a Translational Model for COVID-19 in People with Human Immunodeficiency Virus Infection

**DOI:** 10.3390/microorganisms12071289

**Published:** 2024-06-25

**Authors:** Shoroq Shatnawi, Sachithra Gunasekara, Laura Bashor, Miruthula Tamil Selvan, Mary Nehring, Shannon Cowan, Jerry Ritchey, Susan VandeWoude, Brianne Taylor, Craig Miller, Jennifer M. Rudd

**Affiliations:** 1 Department of Veterinary Pathobiology, College of Veterinary Medicine, Oklahoma State University, Stillwater, OK 74078, USA; shoroq.shatnawi@okstate.edu (S.S.);; 2 Department of Microbiology, Immunology, and Pathology, College of Veterinary Medicine and Biomedical Sciences, Colorado State University, Fort Collins, CO 80521, USA

**Keywords:** human immunodeficiency virus (HIV), SARS-CoV-2, COVID-19, feline immunodeficiency virus (FIV), coinfection, feline

## Abstract

People living with human immunodeficiency virus (PLWH) are a significant population globally. Research delineating our understanding of coinfections in PLWH is critical to care for those navigating infection with other pathogens. The recent COVID-19 pandemic underscored the urgent need for studying the effects of SARS-CoV-2 infections in therapy-controlled and uncontrolled immunodeficiency viral infections. This study established the utility of a feline model for the in vivo study of coinfections. Domestic cats are naturally infected with SARS-CoV-2 and Feline Immunodeficiency Virus, a lentivirus molecularly and pathogenically similar to HIV. In this study, comparisons are made between FIV-positive and FIV-negative cats inoculated with SARS-CoV-2 (B.1.617.2.) in an experimental setting. Of the FIV+ cats, three received Zidovudine (AZT) therapy in the weeks leading up to SARS-CoV-2 inoculation, and two did not. SARS-CoV-2 viral RNA was quantified, histopathologic comparisons of respiratory tissues were made, and T-cell populations were analyzed for immune phenotype shifts between groups. CD4+ T lymphocyte responses varied, with FIV+-untreated cats having the poorest CD4+ response to SARS-CoV-2 infection. While all cats had significant pulmonary inflammation, key histopathologic features of the disease differed between groups. Additionally, viral genomic analysis was performed, and results were analyzed for the presence of emerging, absent, amplified, or reduced mutations in SARS-CoV-2 viral RNA after passage through the feline model. Positive selection is noted, especially in FIV+ cats untreated with AZT, and mutations with potential relevance were identified; one FIV+-untreated cat had persistent, increasing SARS-CoV-2 RNA in plasma five days post-infection. These findings and others support the utility of the feline model for studying coinfection in people with HIV and highlight the importance of antiretroviral therapy in clearing SARS-CoV-2 coinfections to minimize transmission and emergence of mutations that may have deleterious effects.

## 1. Introduction

According to the Joint United Nations Programme on HIV/AIDS (UNAIDS), an estimated 39 million people are living with Human Immunodeficiency Virus (HIV) infection globally as of 2022. Of these, 29.8 million HIV-infected people (76%) have access to a treatment regimen of antiretroviral therapy (ART), leaving about 10 million people globally who cannot attain the resources and medications they need to improve their clinical outcomes [1]. When taken as prescribed, antiretroviral therapies have consistently been shown to result in undetectable viral loads, which also results in people living with HIV (PLWH) living longer, higher-quality lives with little to no risk of transmission to HIV-negative partners. As per HIV.gov [2], certain regions of the globe are disproportionately affected; in particular, this includes large regions of Africa and parts of Asia and the Pacific. These same regions are disproportionately affected by other diseases, food insecurity, and political unrest. Unfortunately, the COVID-19 pandemic, caused by Severe Acute Respiratory Syndrome Coronavirus-2 (SARS-CoV-2), further disrupted access to HIV diagnostics, therapies, and resources.

It is well established that PLWH are at higher risk of respiratory infections due to HIV-targeting CD4^+^ T lymphocytes, resulting in deficiencies of the immune response to infection. Coinfection with SARS-CoV-2 in PLWH quickly resulted in concerns regarding the syndemic nature of these viral infections and the potential for HIV-infected people to have worsened severity of disease, higher rates of hospitalization, and reduced efficacy of vaccine-induced humoral and cell-mediated immunity. Additionally, PLWH posed a complicated question as to the potential role of immune deficiency in variant emergence and prolonged transmission of SARS-CoV-2. During the global COVID-19 pandemic from December 2019 through March 2024, 259 million cases were diagnosed in 223 countries, and more than 7 million people died [3]. In this time, an estimated 1.3 people were newly infected with HIV in 2022, and 630,000 people died from acquired immunodeficiency syndrome (AIDS)-related illness in that same year [1]. Due to mounting coinfection concerns, in 2020, the Centers for Disease Control and Prevention (CDC) classified HIV-infected people at increased risk of being severally ill if coinfected with SARS-CoV-2, especially if they are (1) over the age of 65, (2) diagnosed with an additional comorbidity, (3) diagnosed with AIDS, or (4) untreated for their HIV infection [4]. 

Concerns regarding COVID-19 in PLWH were validated as data from the COVID-19 pandemic underscored the risk coinfection poses in clinical outcomes. In 2022, Dawang et al. published a systematic meta-analysis of clinical outcomes for PLWH presenting with SARS-CoV-2 infection. In this analysis, they found that PLWH is at higher risk for hospitalization with SARS-CoV-2 coinfection [5]. Additional studies have independently associated HIV infection with increased mortality in COVID-19 patients [6,7]. The increased risk for hospitalization and death in PLWH who are subsequently infected with SARS-CoV-2 is especially noted in patients who are not stabilized with ART or do not have access to ART for their HIV [5,8,9]. The importance of ART in coinfection outcomes is paramount, and numerous studies now indicate that PLWH who are adequately treated with ART and have access to basic needs such as shelter and food security are not necessarily at higher risk of SARS-CoV-2 infection [10,11,12], although studies continue to indicate variable results [13,14]. However, PLWH who have reduced CD4+ T cells and compromised cellular immunity due to inadequate or unavailable ART are consistently at increased risk [15]. This immunological diversity, compounded by social and economic confounders among PLWH, complicates studies and highlights the need for an effective, workable animal model that can serve a translational role in studying COVID-19 in PLWH.

There are few animal models that closely mimic both acute COVID-19 and HIV within a single host, so challenges exist in establishing a translational model to best study the immune effects and potential therapeutic or preventive interventions for coinfection in people. Previous studies have shown the domestic cat as a good translational model for COVID-19 in people, as cats are naturally infected with the virus and have been shown to develop diseases very similar to acute COVID-19 in experimental and natural settings [16]. Domestic cats are also naturally infected with an immunodeficiency virus closely related to HIV called Feline Immunodeficiency Virus (FIV). Just like HIV, FIV is a member of the lentivirus genus belonging to the *Retroviridae* family. As compared with HIV, FIV is structurally similar, shares similar genomic organization, has matched lymphocyte tropism, and mimics the pathogenesis of HIV infection in humans [17]. Since 1987, FIV has been applied as a translational model to study HIV [18,19,20,21,22]. FIV naturally infects cats and causes a progressive immune disease characterized by CD4+ T cell depletion, oral infections, and central nervous system involvement [21,23,24,25]. Consequently, domestic cats offer a unique advantage for studying HIV infections, facilitating in-depth investigations of the disease pathogenesis and an animal model for the application of the preclinical evaluation of therapeutics and vaccines [23,26,27]. 

The US Food and Drug Administration (FDA) approved the administration of Zidovudine (3′-azido-2′,3′-dideoxythymidine), also known as AZT, as the first drug to treat HIV patients in 1987 [28]. Zidovudine is utilized in FIV research and is proven to effectively control FIV infection in a similar manner to its effects in HIV [29]. This drug has important translational potential for studying both FIV and HIV because (1) Zidovudine is one of the Nucleoside Reverse Transcriptase Inhibitors (NRTI), which has been shown to inhibit HIV as well as other lentiviruses, including FIV [30], (2) other antiretroviral treatments have not been studied in vivo or have previously demonstrated insufficient efficacy or safety concerns in cats [31,32]. Previous studies evaluated the in vivo administration of Zidovudine in FIV-infected cats and determined that Zidovudine effectively reduces plasma FIV viral load, improves the immunologic and clinical status of FIV-infected cats, increases quality of life, and prolongs life expectancy [33,34,35]. 

To our knowledge, no published studies have evaluated the domestic cat as a translational animal model to study the coinfection of HIV and SARS-CoV-2. In this study, we propose this animal model and evaluate its potential for studying SARS-CoV-2 coinfection in PLWH. Furthermore, we hypothesize that administration of Zidovudine will protect FIV-infected cats from severe clinical outcomes through reduction of both FIV and SARS-CoV-2 viral loads, reduced SARS-CoV-2 viral shedding, and reduced damage to the pulmonary tissue following SARS-CoV-2 infection in FIV-negative and Zidovudine-treated FIV-positive cats. Establishing this model provides valuable insight into the immune responses observed in PLWH who experience SARS-CoV-2 coinfection as well as the importance of ART to support the immune response prior to being infected with SARS-CoV-2. Finally, this study provides a preliminary look at the potential for viral mutations that may contribute to variant emergence not only as a model for human infection but also as a means to laying the groundwork for the cat as a potential animal reservoir for SARS-CoV-2.

## 2. Materials and Methods

### 2.1. Animal Source and Care and Feline Immunodeficiency Viral Infection

Seven (*n* = 7) specific pathogen-free cats (4 females and 3 males, ages ranging from 12 to 16 months) were obtained from an accredited cat colony at Colorado State University (CSU) for use in this study. Complete baseline characteristics are available in Supplementary Table S1. Cats were housed at an AAALAC-international accredited animal facility at CSU, where they were inoculated with FIV based on CSU’s IACUC-approved protocols. To establish FIV infection in cats, five (*n* = 5) domestic cats (*Felis silvestrus catus*) were infected with FIV twelve months prior to beginning Zidovudine (AZT) or vehicle therapy. Briefly, cats were inoculated through intravenous and oral routes with 1 mL of FIVc36 viral stock diluted 1:80 in 0.9% saline, which has previously been validated to demonstrate acute immunopathogenic effects and reproducible high FIV titers [25,36]. FIV infection was confirmed by applying droplet digital polymerase chain reaction (ddPCR) from plasma samples from the FIV-infected cats at CSU prior to transportation. FIV-negative cats (*n* = 2) were sham-inoculated with 1 mL of 0.9% saline via the same inoculation procedure [37]. Of the seven cats acquired from CSU for this study, five (*n* = 5) were FIV-infected, and two (*n* = 2) were confirmed as FIV-negative prior to transportation. Upon arrival, animals were individually housed within Animal Biosafety Level 3 (ABSL-3) barrier animal rooms at Oklahoma State University, an AAALAC International accredited animal facility, and fed dry and wet food with access to water ad libitum. Animals were allowed 21 days of acclimation prior to starting AZT or vehicle therapy. All cats were subcutaneously implanted in the dorsum with temperature-sensing microchips (Bio Medic Data Systems, Seaford, DE, USA) prior to SARS-CoV-2 inoculation. Baseline body weights, temperatures, clinical evaluations, and nasal swab sampling were obtained prior to initiating AZT or vehicle therapy. All animals were in apparent good health after transportation and prior to the study onset (see institutional review board statement below).

### 2.2. SARS-CoV-2 Viral Source, Propagation, and Inoculation

SARS-CoV-2 virus isolate CoV19/USA/PHC658/2021 (Delta Variant), obtained from BEI Resources (Manassas, VA, USA), was passaged 6 times in Vero E6 cells in Dulbecco’s Modified Eagle Medium (Gibco, Carlsbad, CA, USA), which contained 5% Fetal Bovine Serum (Hyclone, Logan, UT, USA), and 1% Penicillin–Streptomycin at 37 °C. The titration and quantification of the virus was performed by using Vero E6 cells through the application of the standard MERS-CoV quantification assay [9] with subsequent utilization of the Reed and Muench method [38] to calculate viral TCID_50_ as previously described [16].

### 2.3. Experimental Design and Viral Challenge

**Figure 1:**
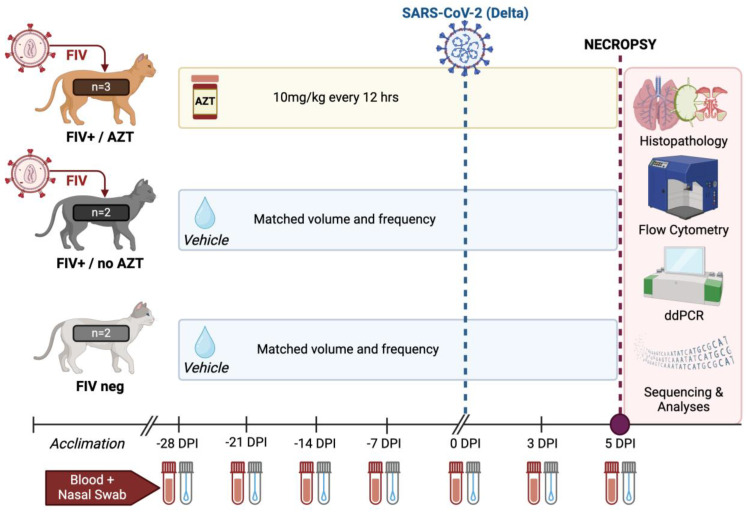
Experimental design. This figure summarizes the experimental design utilized throughout this study and outlines distribution of cats and treatment assignments. FIV = Feline Immunodeficiency Virus; AZT = 3′-azido-2′,3′-dideoxythymidine (Zidovudine).

This study lasted a total of 33 days and included a total of *n* = 7 cats (*n* = 5 FIV-infected; *n* = 2 FIV-negative). FIV-infected cats were not treated with any antiretroviral therapy prior to arrival at Oklahoma State University, and all were apparently healthy at the time of study initiation. After an acclimation period, Zidovudine (henceforth referred to as AZT) was initiated in three FIV-infected cats. AZT (50 mg/mL) was administered at 10 mg/kg every 12 h by mouth for 28 days. All animals not in the treatment group (*n* = 4) received equivalent volumes of vehicle by mouth. After 28 days of AZT or vehicle administration, all cats (*n* = 7) were intratracheally and intranasally inoculated with SARS-CoV-2. In brief, cats were anesthetized with ketamine (4 mg/kg) (Covetrus, Dublin, OH, USA), dexmedetomidine (20 μg/kg) (Orion, Espoo, Finland), and butorphanol (0.4 mg/kg) (Zoetis, Girona, Spain) intramuscularly. Cats were positioned in ventral recumbency and intubated so that the end of an endotracheal tube was positioned within the distal trachea as described previously [16]. Using a 3 cc syringe, 3 × 10^5^/kg SARS-CoV-2 (Delta Variant) was inoculated intratracheally, followed by 4 × 10^5^/cat intranasally (0.5 ml split between 2 nostrils). Six days after SARS-CoV-2 inoculation, all cats were culled for final necropsy and tissue collection. This is visually outlined in Figure 1.

### 2.4. Clinical Evaluation

Animals were observed for any clinical abnormalities and evidence of morbidity at least once daily prior to SARS-CoV-2 inoculation and at least twice daily after SARS-CoV-2 inoculation by a licensed veterinary general practitioner. Clinical evaluation was performed utilizing a clinical scoring system previously described [16], and presented with minor modifications in the included table. In brief, clinical evaluation included monitoring the body weight, systemic temperature, activity levels, behavioral changes, respiratory effort, presence of ocular or nasal discharge, and observed coughing or wheezing with scores assigned as described in the scoring table [16]. Each clinical parameter was categorized as 0, 1, 2, or 3, where 0 indicated normal or healthy, 1 was mild, 2 was moderate, and 3 was marked changes in the clinical observations or assessments. This scoring is further outlined in Table 1. Respiratory rate, activity levels, and behavior change were assessed prior to handling or stimulation. Oxygen saturation (SpO_2_) was measured via pulse oximetry on the ear. Clinical parameters were additionally summated in order to assign individual animals a total clinical score every 24 h throughout the duration of the study.

### 2.5. Sample Collection

Blood samples and nasal swabs were collected under light sedation as previously described [16]. Blood was collected from all cats (*n* = 7) every 7 days through AZT or vehicle therapy, 28 days prior to SARS-CoV-2 infection (−28 DPI) through SARS-CoV-2 infection (0 DPI), as well as at days 3 and 5 post-SARS-CoV-2 inoculation (3 DPI, 5 DPI). Blood samples (no more than 4 mL per timepoint) were collected via jugular venipuncture and immediately processed for varied analyses and SARS-CoV-2 viral quantification. Nasal swabs were collected from both nares post-SARS-CoV-2 inoculation (0 DPI, 3 DPI, and 5 DPI) by using ultra-fine flocked swabs (Puritan, Guilford, ME, USA) and then placed in 2 mL tubes containing 200 µL RNAlater solution (Sigma, St. Louis, MO, USA) and stored at −80 °C for laboratory analysis. Details for these sample collections are as previously described [16]. Blood from −28 DPI (28 days prior to SARS-CoV-2 inoculation) and blood and nasal swabs from 0 DPI were used as baseline measurements for downstream analyses. At 5 DPI, all animals were humanely euthanized with pentobarbital (>80 mg/kg IV) and necropsied to collect tissue samples as previously described [16]. Tissues collected were kept in formalin for histologic examination or fresh frozen for RNA analysis, as described below.

### 2.6. FIV and SARS-CoV-2 Viral RNA Analyses

Viral quantification was performed to assess FIV loads as well as SARS-CoV-2 viral loads. For FIV, the QIAmp Viral RNA Mini Kit (Qiagen, Germantown, MD, USA) was used to extract FIV RNA from collected plasma, according to the manufacturer’s protocols. This was followed synthesis of cDNA using Superscript II reverse transcriptase (Invitrogen, Carlsbad, CA, USA) in individual reactions with random hexamers (Invitrogen). cDNA was treated with RNase Out (Invitrogen) prior to droplet digital PCR (ddPCR) quantification and as previously described [39]. ddPCR was performed to quantify FIV viral loads using 500 nM FIV-cgag primers and 250 nM probe [40]. The selected primer and probe sequences are as follows: forward primer 5′-CTACACTCTTTACACGTTTGTG-3′; reverse primer 5′-AGGAGTATACTGGCATTTCG-3′, probe 5′-GGTGTTATAATGGCCCTTGCAAACTC

A-3′. The PCR reaction mixture contained 15.75 μL of ddPCR master mix, 1 μL FIV-cgag primer and 1 μL ddPCR Supermix for Probes (No dUTP) (Bio-Rad, Hercules, CA, USA), and 5.25 µL cDNA template. Duplicate 20 μL samples of this mixture were partitioned using a QX200 droplet generator (Bio-Rad Laboratories, Inc.) to respective wells of DG8^TM^ cartridges (Bio-Rad Laboratories, Inc. Hercules, CA, USA) with 70 µL of droplet generation oil for probes (Bio-Rad Laboratories, Inc. Hercules, CA, USA) and processed in a C1000 Touch Thermal Cycler (Bio-Rad) using the following cycling protocol: 95 °C for 10 min for initial denaturation, 94 °C for 30 s, 61 °C for 60 s for annealing and extension for 45 cycles, 98 °C for 10 min for enzyme deactivation, and 4 °C for 30 min for droplet stabilization, followed by an indefinite 4 °C hold.

SARS-CoV-2 viral RNA load was extracted from plasma and nasal swab samples using QIAamp Viral RNA Mini Kit (Cat. No./ID: 52906) and from tissue using RNeasy kit (Qiagen, Cat. No./ID: 74106) after homogenizing the tissue with 600 µL RLT (lysis buffer) in the tissue homogenizer. Next, cDNA was synthesized using Superscript II reverse transcriptase (Invitrogen, Carlsbad, CA, USA) in individual reactions with random hexamers (Invitrogen) and subsequently treated with RNase Out (Invitrogen) [39], followed by ddPCR [41], performed to quantify SARS-CoV-2 viral loads. ddPCR was performed using N1 and N2 primers (500 nM each) and probe (at 250 nM) (Cat. No. 10006713, Integrated DNA Technologies, Inc., Coralville, IA, USA). The reaction mixture contained the following: 1.5 μL of N1, 1.5 μL of N2, 10 μL of ddPCR Supermix for Probes (No dUTP) (Bio-Rad, Hercules, CA, USA), and 6.3 μL of cDNA template. Droplets were generated using a QX200 droplet generator (Bio-Rad) by duplicate 20 μL samples, which were partitioned as previously described [16]. Samples were then processed in a C1000 Touch Thermal Cycler (Bio-Rad) according to this protocol: 95 °C for 10 min for initial denaturation, 95 °C for 30 s, 55 °C for 60 min for annealing and extension for 45 cycles, and 98 °C for 10 min for enzyme deactivation. A ramp rate of 2 °C/s was used throughout the protocol. Positive controls, viral RNA extracted from SARS-CoV-2 Delta Variant viral stock and diluted 1:15,000, negative controls, and no template control (NTC) were included in each run [16]. Amplified samples were read in the FAM and HEX channels of a QX200 reader (Bio-Rad). Data were analyzed and expressed as Log10 (copies/mL) with Quanta soft Software 1.7 (Bio-Rad) [16].

### 2.7. Flow Cytometry

Flow cytometry aimed to evaluate shifts in immune T cell subtypes, including CD4+, CD4:CD8 ratios, and CD8+. Briefly, blood was collected in EDTA tubes and 50 μL of collected blood was treated with mouse monoclonal antibodies directed to feline markers for CD4 antigens (Fisher, Hampton, NH, USA, clone 3-4F4, FITC), CD8 antigen (Southern Biotech, Birmingham, AL, USA, clone fCD8, PE), and CD21 (Bio-Rad, CA2.1D6, AF647) according to the manufacturer’s recommended volume per test. Samples were then kept in the dark for 20 min at 4 °C. Red blood cells were lysed, and samples were fixed using the TQ-Prep Workstation and IMMUNOPREP Reagent System (Beckman Coulter Inc, Brea, CA, USA). The control samples, including unstained, single-stained, and pertinent Flourescence Minus One (FMO) gating controls, were prepared for each experiment. Data was obtained using BD FACSDiva™ Software Version 9.0.1 (BD Biosciences, San Jose, CA, USA) interfaced with a BD FACSAria^TM^ SORP instrument (BD Biosciences, San Jose, CA, USA). Compensation values were determined in the FACSDiva software using single stained controls. Gating was determined from singlets to lymphocytes to CD4 and CD8. The percentages of lymphocyte subsets positive for each marker were evaluated over time and compared to baseline values and between treatment groups to assess for alternations in lymphocyte immunophenotypes.

### 2.8. Histopathology 

Necropsy was conducted on all cats (*n* = 7) at 5 DPI of the study. As per the protocol outlined in a previous study [16], all tissues were systematically collected from pre-determined locations by a board-certified veterinary pathologist. Collected tissues include lung tissues (right cranial, right middle, and right caudal lobes), nasal turbinate, distal trachea, and tracheobronchial lymph nodes, and all were divided and placed into either 1 mL tubes and frozen at −80 °C or placed into standard tissue cassettes which were then formalin-fixed in 10% neutral-buffered formalin for 96 h. After 96 h, formalin-fixed tissues were transferred to 70% ethanol for 72 h. Formalin-fixed tissues were then trimmed, processed, and prepared for histopathology as previously described [16,39]. All tissue sections were embedded in paraffin, trimmed in 5 μm sections, collected onto charged slides, and stained with hematoxylin and eosin (H&E) for assessment with light microscopy. Necropsy tissues were evaluated for histopathologic lesions reported in humans with COVID-19 [42,43,44,45] and as per previous studies in SARS-CoV-2 infected cats [16]. Pulmonary tissues were assessed as described previously [46]. In brief, pulmonary tissues were assessed and scored for alveolar damage (e.g., pneumocyte necrosis and hyaline membrane formation), serous edema, alveolar fibrin deposition, alveolar histiocytosis, perivascular infiltrates, type II pneumocyte hyperplasia, peri-bronchial inflammation, smooth muscle hyperplasia, thrombosis, and fibrinoid vasculitis. Tissues from nasal turbinates were evaluated for ulceration, goblet cell hyperplasia, and mucosal or submucosal inflammation. All tissues were assigned a quantitative histopathological score from 0 to 4 based on previously documented standards [16,39,47], with 0 = no apparent pathology or change; 1 = minimal change (minimally increased numbers of inflammatory cells); 2 = mild change (mild inflammatory infiltrates, alveolar damage/necrosis, fibrin deposition and/or exudation); 3 = moderate change (as previously described but more moderately extensive); 4 = marked changes (as previously described but with severe inflammation, alveolar damage, hyaline membrane formation, necrosis, exudation, vasculitis, and/or thrombosis). Scoring encompasses the extent of lesions across collected tissue sections, with more severe scores also indicating more extensive damage. All tissues were assessed and scored by a board-certified veterinary pathologist who was blind to study groups to eliminate bias and to ensure scientific accuracy and rigor.

### 2.9. Next-Generation Sequencing and Analyses

RNA samples were used to generate complementary DNA (cDNA), which was used as input for tiled amplicon PCR enrichment and library preparation. Reverse transcription was carried out in duplicate with 5 μL of RNA using SuperScript II RT (Thermo Fisher Scientific, Waltham, MA, USA) and frozen at −20 °C prior to next steps. SARS-CoV-2 genomic material was amplified using the ARTIC version 4 (V4) primer scheme designed by the ARTIC Network [48]. This multiplex PCR generates ~400 bp overlapping (“tiled”) amplicons that span the full viral genome. ARTIC primer sequences are publicly available here: https://github.com/artic-network/artic-ncov2019/tree/master/primer_schemes/nCoV-2019 (accessed on 4 August 2023). PCR products were run on a 1% agarose gel and quantified with a Qubit dsDNA 1× High-Sensitivity Assay kit (Thermo Fisher Scientific). Sequencing libraries were prepared in technical duplicate, and 100 ng DNA was used as input for the NEBNext Ultra II DNA Library Prep kit. Replicate samples were individually indexed with NEBNext Multiplex Oligos for Illumina (New England Biolabs, Ipswich, MA, USA). Ampure XP beads (Beckman Coulter) were used for bead cleanups and a single 0.65X size selection after adapter ligation. Libraries were pooled evenly for sequencing with a v2 500 cycle (2 × 250) bp kit (Illumina, San Diego, CA, USA). The sequencing run was carried out on an Illumina MiSeq instrument at the Colorado State University Next Generation Sequencing Facility. Sequencing data were analyzed with a custom bioinformatics pipeline designed to call single nucleotide and structural variants in viral populations [49] available here: https://github.com/stenglein-lab/viral_variant_caller (accessed 4 August 2023). Sequencing reads were trimmed for quality and adapters and aligned to the Wuhan-Hu-1 reference sequence (GenBank MN908947.3). Low-frequency variant calling was carried out with a minimum depth of coverage cutoff of 40× and minimum allele frequency cutoffs of 0.1% and 3%. Variant call format (vcf) files output by the pipeline were used as input for the SNPGenie pipeline to calculate nonsynonymous and synonymous nucleotide diversity at the within-host population level and by gene product [50]. Data visualizations were prepared in R Statistical Software, Version 4.3.3 [51].

### 2.10. Statistical Analyses

For statistical analysis, results were analyzed using Graph Pad Prism 9.0 Software (La Jolla, CA, USA) and presented as the mean ± SEM when applicable. Two-way ANOVA, Kruskal–Wallis test, and repeated measures ANOVA with Tukey post hoc analysis were used to compare differences in FIV and SARS-CoV-2 viral load in plasma and tissue in coinfection-treated and coinfection-untreated groups between sample types, for each tissue individually, and between tissues. *p*-values less than 0.05 were considered statistically significant.

## 3. Results

### 3.1. FIV Viral RNA Expression Is Confirmed in Plasma of Previously Infected FIV+ Cats

**Figure 2:**
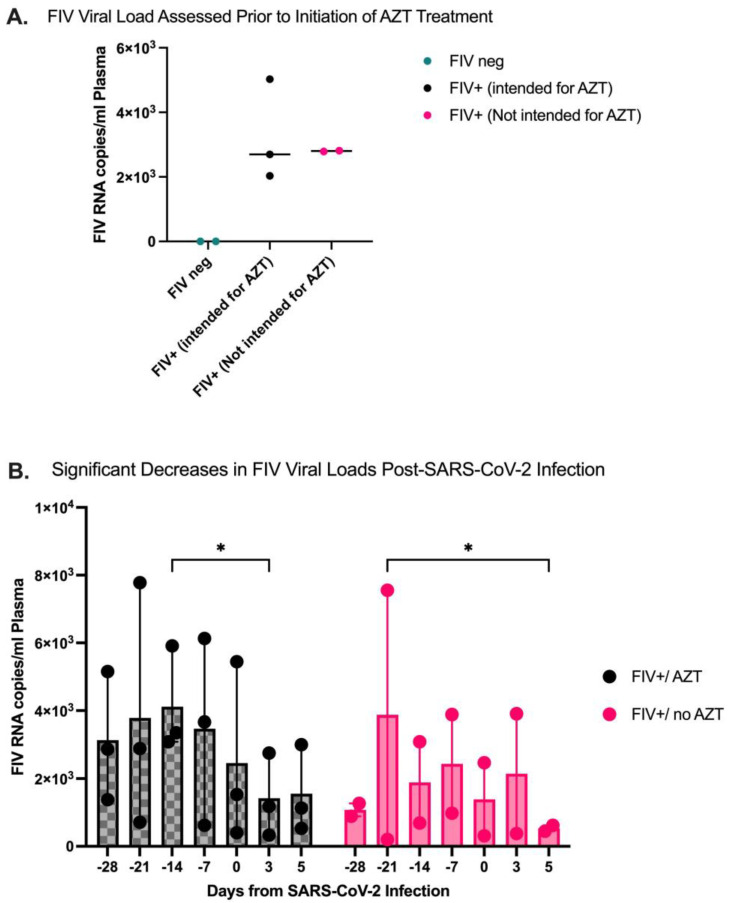
FIV viral quantification in plasma pre- and post-infection with SARS-CoV-2. AZT or vehicle was initiated at −28 DPI. SARS-CoV-2 inoculation occurred at 0 DPI. (**A**) FIV viral RNA was quantified in all cats prior to transportation to OSU. Uninfected cats were confirmed negative for FIV viral RNA in plasma, and FIV-infected cats were confirmed positive for viral RNA in plasma. (**B**) FIV viral load was quantified in plasma weekly in all cat groups through AZT or vehicle therapy. FIV viral RNA was significantly decreased in cat group FIV+/AZT between −14 DPI and 3 DPI (*p* = 0.044). FIV viral RNA in plasma for FIV+/no AZT cats showed significant decreases from −21 DPI to 5 DPI (*p* = 0.042). Statistical comparisons are made via two-way ANOVA. * *p* < 0.05. No FIV viral load was detected in FIV-negative cats at any point in the study.

Prior to SARS-CoV-2 inoculation or the initiation of Zidovudine (AZT) treatment, FIV-infected cats (*n* = 5) were evaluated for FIV RNA expression in plasma. FIV infection was confirmed in FIV+ cats (Figure 2A), and cats were randomly selected for AZT treatment (*n* = 3) or Vehicle treatment (*n* = 2). FIV RNA load was additionally quantified during AZT or vehicle therapy, which was established over 28 days, with blood samples collected every 7 days for the evaluation of FIV viral RNA copies per mL of plasma. A significant decrease in FIV viral load was seen in both FIV-infected groups after SARS-CoV-2 inoculation, with a significant decrease in FIV+/AZT between −14 DPI and 3 DPI (*p* = 0.044) and a significant decrease in FIV+/no AZT between −21 DPI and 5 DPI (*p* = 0.042) (Figure 2B).

### 3.2. AZT-Treated FIV-Infected Cats Have Significant Increases in CD4+ Cells Post SARS-CoV-2 Infection

**Figure 3:**
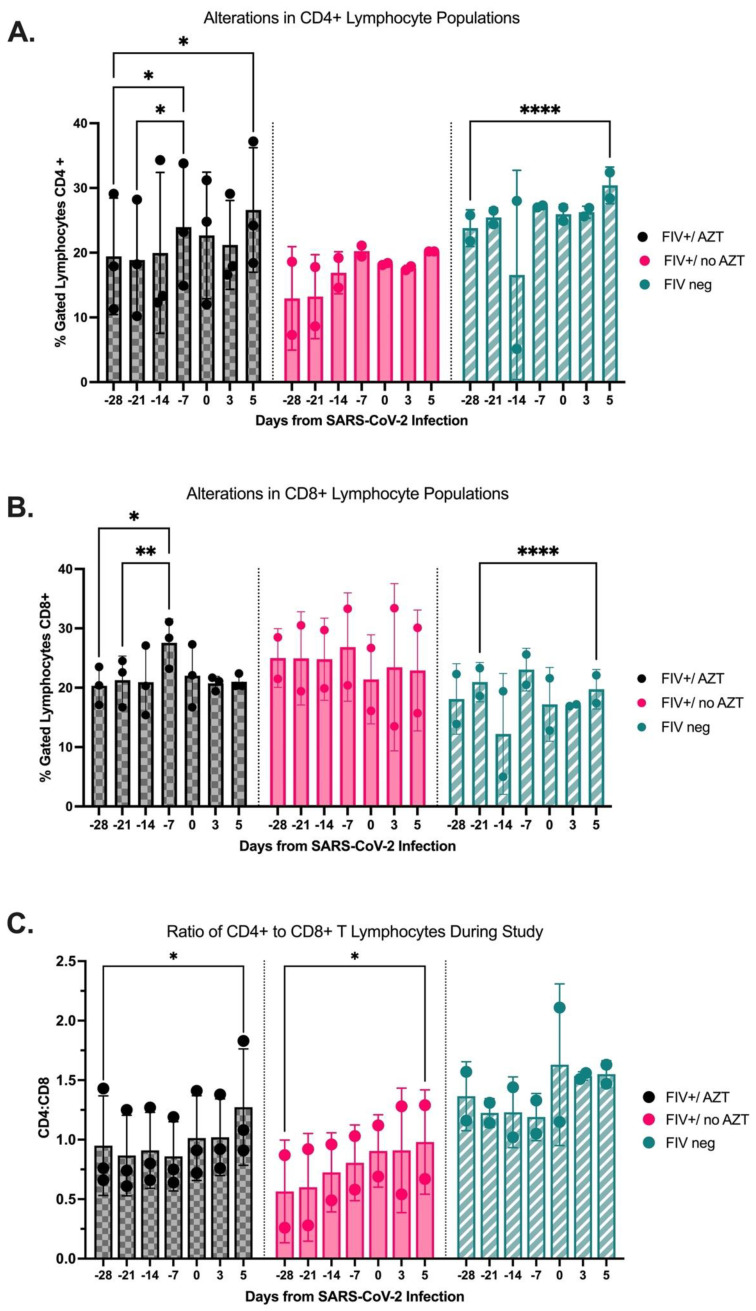
Alterations in lymphocyte immunophenotypes using flow cytometric analysis. (**A**) CD4+ lymphocytes; %CD4+ of total lymphocytes was measured using flow cytometry. %CD4+ is significantly increased in FIV+/AZT group from day −28 through 5 DPI as well as in the FIV-negative group through this same time frame (*p* = 0.0207, *p* = 0.0001 respectively). (**B**) CD8+ T cells. Significant increase in %CD8+ is noted in FIV+/AZT group at day −7 compared to day −28 (*p* = 0.0250). In contrast, a significant decrease in %CD8+ is seen in FIV-negative cats from day −21 to 5 DPI (*p* = 0.0001). No significant change in CD4+ or CD8+ populations is noted in the FIV+-untreated group. (**C**) CD4:CD8 ratios are significantly increases in FIV-infected cats through the study duration as compared with FIV-negative cats. FIV+/AZT (*n* = 3); FIV+/no AZT (*n* = 2); FIV-negative (*n* = 2); statistical comparisons are made via mixed-effect analysis and two-way ANOVA. **** *p* < 0.0001; ** *p* < 0.01; * *p* < 0.05.

Flow cytometry was used to evaluate the immunophenotype shifts of lymphocytes in blood throughout the study. Lymphocytes were gated, and subsets were determined based on CD4, CD8, and CD21 surface antigen staining. Percentages of total gated lymphocytes with CD4 or CD8 staining were calculated. Data for evaluating the percentage of CD4+ and CD8+ demonstrate a significant increase in the %CD4+ in FIV+/AZT cats at 5 DPI as compared to day −28 and a significant increase in FIV-negative cats over this same time frame (*p* = 0.0207 and *p* = 0.0001, respectively) (Figure 3A). Percentage CD8+ reveals a significant increase in FIV+/AZT cats from study start through day -7pre-SARS-CoV-2 (*p* = 0.0250) but no significant change post-SARS-CoV-2 infection, whereas FIV-negative cats had a significant reduction in percentage CD8+ lymphocytes from pre-SARS-CoV-2 (−21 DPI) to 5 DPI (*p* = 0.0001) (Figure 3B). No significant changes were seen in either CD4+ or CD8+ subsets for the FIV+/no AZT group. Finally, the CD4:CD8 ratios significantly increased from the study’s start through 5 DPI in the FIV-infected groups but were not significantly changed in the FIV-negative cats (Figure 3C).

### 3.3. Quantification of SARS-CoV-2 Viral RNA in Tissue Indicates Higher Viral Loads in Upper Respiratory Tract in All Infected Cats and Detectable Viral RNA in Plasma of FIV+/no AZT Cat at 5 DPI

**Figure 4:**
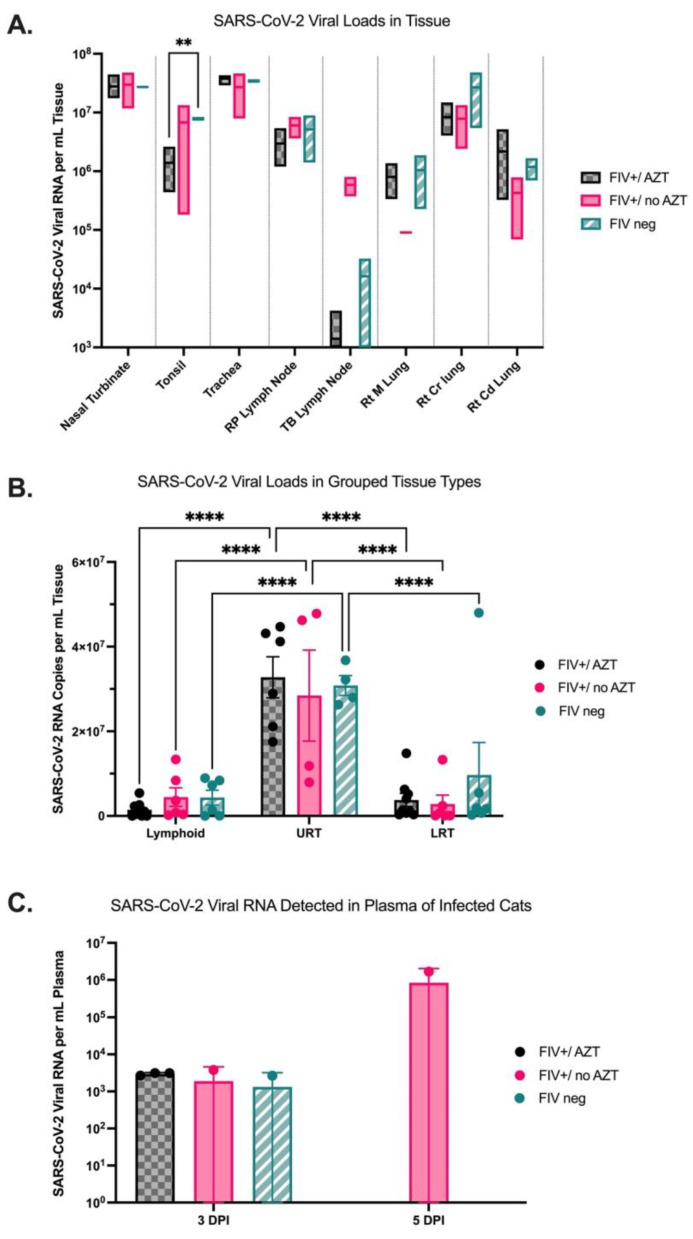
Quantification of SARS-CoV-2 viral RNA from tissues collected at the necropsy 5 DPI. (**A**) SARS-CoV-2 RNA was detected using ddPCR in tissues collected at necropsy 5 DPI, but no significant differences were seen between groups except for a significant increase in RNA from the tonsils in the FIV-negative group as compared with FIV+/AZT (*p* = 0.0058). (**B**) Upper respiratory tract tissues (URT) contain significantly higher SARS-CoV-2 viral loads as compared to lower respiratory tract (LRT) and lymphoid tissues in all cat groups (*p* = 0.0055). (**C**) SARS-CoV-2 viral load was also quantified from plasma using ddPCR with significant drops from 3 to 5 DPI, resulting in no detectable SARS-CoV-2 at 5 DPI in the FIV-negative and FIV+/AZT groups (*p* = 0.0025). While no statistically significant increase is noted between groups, one cat from the FIV+/no AZT group had a persistent detectable viral load in plasma through 5 DPI. FIV+/AZT (*n* = 3); FIV+/no AZT (*n* = 2); FIV-negative (*n* = 2); statistical comparisons are made via mixed-effect analysis and two-way ANOVA, **** *p* < 0.0001,** *p* < 0.01.

Tissues collected at necropsy at 5 DPI were evaluated for SARS-CoV-2 viral RNA per mL of tissue for all groups using ddPCR. Within specific tissue collections, no significant differences were noted between groups except for a significant increase in SARS-CoV-2 viral load in the tonsil of FIV-negative cats as compared with FIV+/AZT-treated cats (*p* = 0.0058) (Figure 4A). Significantly increased SARS-CoV-2 viral loads were detected in upper respiratory tract tissues (nasal turbinates and distal trachea) as compared to lower respiratory tract tissues (right cranial, right middle, and right caudal lung lobes) and lymphoid tissues (tonsils, retropharyngeal lymph nodes, and tracheobronchial lymph nodes) within all three groups (*p* = 0.0055) (Figure 4B). SARS-CoV-2 viral RNA was also quantified from plasma collected at 0, 3, and 5 DPI using ddPCR. No SARS-CoV-2 was detected at 0 DPI in plasma but was detected at 3 DPI in plasma of all three cats from the FIV+/AZT group, one of two cats from the FIV+/no AZT group, and one of two cats from the FIV-negative group. This SARS-CoV-2 significantly dropped to “not detected” at 5 DPI in the FIV-negative and FIV+/AZT groups (*p* = 0.0025). Interestingly, at 5 DPI, only one cat maintained detectable SARS-CoV-2 RNA in plasma, and this cat was from the FIV+-untreated group with over three-fold higher viral load as compared with the 3-DPI load in that same cat (Figure 4C).

### 3.4. Significant Histopathologic Differences between Groups after SARS-CoV-2 Infection

All cats were monitored for clinical abnormalities throughout the study period, as described [16]. Clinical parameters assessed include core temperature, body weight, ocular/nasal discharge, respiratory rate and effort, and alterations in activity or behavioral patterns. Additionally, specific physiological parameters, such as oxygen saturation (SpO_2_%), were assessed. Observations for coughing or wheezing were diligently monitored in all cat groups from 0 DPI to 5 DPI. There were no statistically significant differences in the observable clinical disease between groups throughout the study duration and following the administration of the SARS-CoV-2 inoculum. 

**Figure 5:**
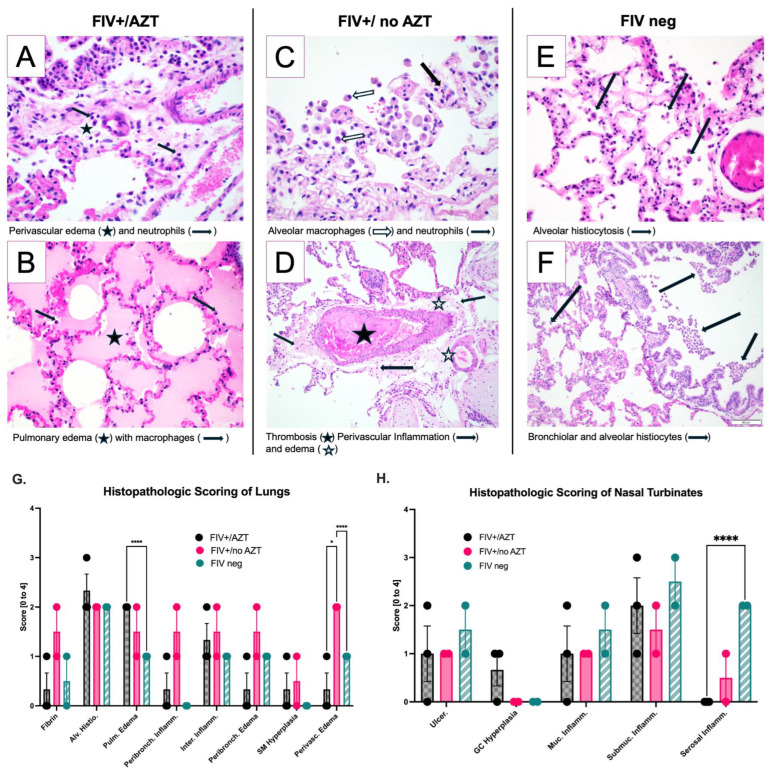
Comparisons of histopathologic disease noted in SARS-CoV-2 infected cats. Lungs from FIV+/AZT cats exhibited minimal to mild interstitial inflammation ((**A**), arrows) and perivascular edema ((**A**), star), mild to moderate alveolar histiocytosis ((**B**), arrows), and mild serous exudate/pulmonary edema ((**B**), star). Lung tissue from FIV+/no AZT cats exhibited moderate alveolar histiocytosis ((**C**), white arrow), minimal to mild serous exudate/pulmonary edema ((**D**), white star), minimal to mild intra-alveolar inflammation ((**C**,**D**) black arrow), and thrombosis ((**D**), black star). FIV-negative cats demonstrated moderate alveolar histiocytosis ((**E**), black arrow), and moderate alveolar and bronchial histiocytosis ((**F**), black arrow). (**G**) summarizes the histopathology scoring of collected lung tissues from all three groups; minimal serous exudate/pulmonary edema is noted in the FIV+/AZT group. FIV-negative cats displayed a significant increase in perivascular edema (Perivasc. Edema) and minimal to no lesions of intra-alveolar inflammation. (**H**) summarizes the nasal turbinate histopathology scoring showing a significant increase in muscular/serosal inflammation in FIV-negative compared to FIV+/AZT. In total histopathology scoring, lungs were assessed for intra-alveolar fibrin (Fibrin), alveolar histiocytosis (Alv. Histio.), serous exudate/pulmonary edema (Pulm. Edema), peribronchial inflammation (Peribronch. Inflamm.), interstitial inflammation (Inter. Inflamm.), smooth muscle hyperplasia (SM Hyperplasia), and perivascular edema (Perivasc. Edema). Nasal turbinates were assessed for ulceration (Ulcer.), goblet cell hyperplasia (GC Hyperplasia), mucosal inflammation (Muc. Inflamm.), submucosal gland inflammation (Submuc. Inflamm.), and muscular/serosal inflammation (Serosal Inflamm.). **** *p* < 0.0001; * *p* < 0.05. scale bar seen on image (**F**) applies to all histology images in this figure.

Histopathologic evaluation of lung and nasal turbinate tissue was performed for all study animals (*n* = 7) by a board-certified veterinary pathologist. Histopathologic assessment for FIV-negative and FIV+/AZT cats had moderate alveolar damage. Figure 5A,B, representing the lung from FIV+/AZT-group cats, contained mild to moderate alveolar histiocytosis, mild serous exudate/pulmonary edema, and minimal to mild interstitial inflammation. C and D characterize the FIV+/no AZT cat group; in this group, findings included moderate alveolar histiocytosis, minimal to mild serous exudate/pulmonary edema, and minimal to mild intra-alveolar inflammation. E and F are FIV-negative/no Tx; findings included moderate alveolar histiocytosis, minimal serous exudate/pulmonary edema, minimal smooth muscle hyperplasia, and minimal to no intra-alveolar inflammation. A histopathologic assessment scoring of pulmonary lesions revealed a significant increase in serous exudate and pulmonary edema in FIV+/AZT-treated cats as compared to FIV-negative cats (*p* < 0.0001), and perivascular inflammation was significantly more evident in FIV+/no AZT compared to other groups (*p* < 0.0001 and *p* < 0.0377) Figure 5G. Nasal turbinate tissues revealed a significant increase in muscular/serosal inflammation in FIV-negative compared to FIV+/AZT (*p*< 0.0001), as shown in Figure 5H. 

### 3.5. Sequencing and Mutation Analyses Reveal Positive Selection Signatures and Mutations after SARS-CoV-2’s Passage through Feline Host

Complete viral genomes were sequenced at a high depth of coverage (median: 3140X) from viral RNA recovered from lung and nasal turbinate tissues, with observed lower coverage in lung tissue samples relative to nasal turbinate. A dropout region in SARS-CoV-2 open reading frame (ORF) 6/ORF7 was present in the viral inoculum and in infected cats. Within-host variants found in the inoculum virus included a total of 46 mutations detected in the SARS-CoV-2 inoculum virus at ≥3% allele frequency and were consistent with Delta Variant lineage B.1.617.2. Five of the mutations in the inoculum virus (at ≥3% frequency) were not found at all in samples from infected cats. Within-host variants found in cats included 131 unique mutations detected in cats at a frequency ≥3%; 55 of these mutations were also present in the inoculum virus at some level (>0.1%). Therefore, the remaining 76 of these mutations were detected in cats but not in the inoculum virus at any level. We identified <15 mutations in most cat samples, but one sample had 29. Of these 76 sites of interest, we annotated and predicted the effects of mutation. Seventy-two were single-nucleotide variants (SNVs) and four were indels (all causing frameshifts). Predicted effects of SNVs included missense (*n* = 82), stop gained (*n* = 2), and synonymous (*n* = 31); effects of indels included frameshift (*n* = 3) and frameshift variant and stop gained (*n* = 1).

#### 3.5.1. Assessment of Variant Numbers in Tissue and Treatment Type

**Figure 6:**
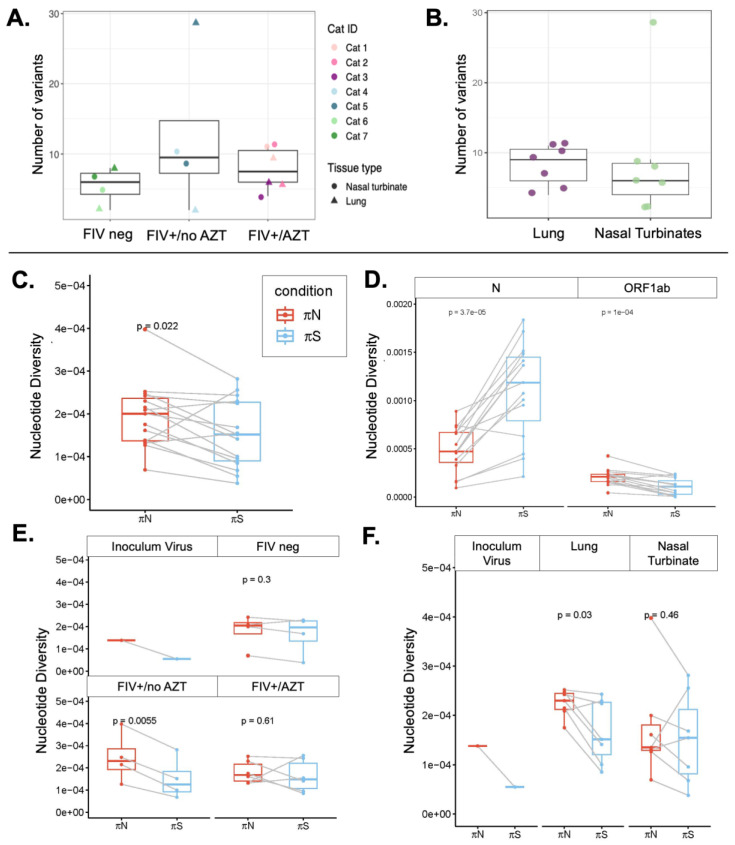
(**A**) Each point indicates the number of variants detected at ≥3% allele frequency in SARS-CoV-2 genome from each cat sample. There was no significant difference in the number of within-host variants among treatment groups (ANOVA, df = 2, *p* = 0.419). (**B**) Each point indicates the number of variants detected at ≥3% frequency in SARS-CoV-2 genome from each cat sample. There was no significant difference in the number of within-host variants between tissue types (*t*-test, df = 6, *p* = 0.4803). (**C**–**F**) Signatures of positive (πN > πS) and purifying (πN < πS) selection are observed in SARS-CoV-2 genomes from different cat samples and groups: (**C**) the investigation of selection through the comparison of nonsynonymous (πN) and synonymous (πS) nucleotide diversity revealed that measurements of πN in our dataset were significantly greater than πS at the within-host population or whole-genome level (*t*-test: t = 2.5828, df = 14, *p*-value = 0.02169). (**D**) SARS-CoV-2 ORF1ab showed signs of positive selection (*p*-value = 0.00001), while a significant signature of purifying selection was observed in the nucleocapsid (N) gene (*p*-value = 0.000037). (**E**) FIV+/no Tx group had a signature of positive selection as compared to other cat groups (*p*-value = 0.0055. (**F**) Comparing between tissue types, lung samples demonstrated signatures of positive selection overall (*p*-value = 0.03).

There was no significant difference in the number of within-host variants among treatment groups (ANOVA, df = 2, *p* = 0.419) (Figure 6A). Also, there was no significant difference in the number of within-host variants between the two tissue types: lung and nasal turbinate (*t*-test, df = 6, *p* = 0.4803) (Figure 6B).

#### 3.5.2. Signature of Selection

The investigation encompassed an assessment of nonsynonymous (πN) and synonymous (πS) nucleotide diversity to explore within-host diversity and discern signatures of selection. Overall, we see a signature of positive selection in cat lung and nasal turbinate samples. It was observed that πN values in our dataset were of significantly higher value than πS values overall, indicating a positive selection phenomenon (*t*-test: t = 2.5828, df = 14, *p* = 0.02169). Among the 15 samples analyzed, including the inoculum, three cat samples demonstrated purifying selection (πS > πN) (Figure 6C). 

A noteworthy finding surfaced in the context of the cat group classification, where positive selection was observed in the FIV+/no Tx group, displaying significantly greater πN than πS relative to other cat groups (*p* = 0.0055) (Figure 6D). Furthermore, the search for selection signatures extended to tissue-level examination, revealing the lung to harbor positive selection (πN > πS) with a *p*-value of 0.03 (Figure 6E). Delving into the gene-level analysis, ORF1ab exhibited evidence of positive selection (πN > πS) with a significant *p*-value of 0.00001, while the nucleocapsid (N) gene displayed significant purifying selection (πN < πS) with a *p*-value of 0.000037 (Figure 6F). At the gene level, an overall signature of positive selection was observed in ORF1ab, but purifying selection was observed in the N gene.

#### 3.5.3. Variants Observed in SARS-CoV-2 Genomes

**Figure 7:**
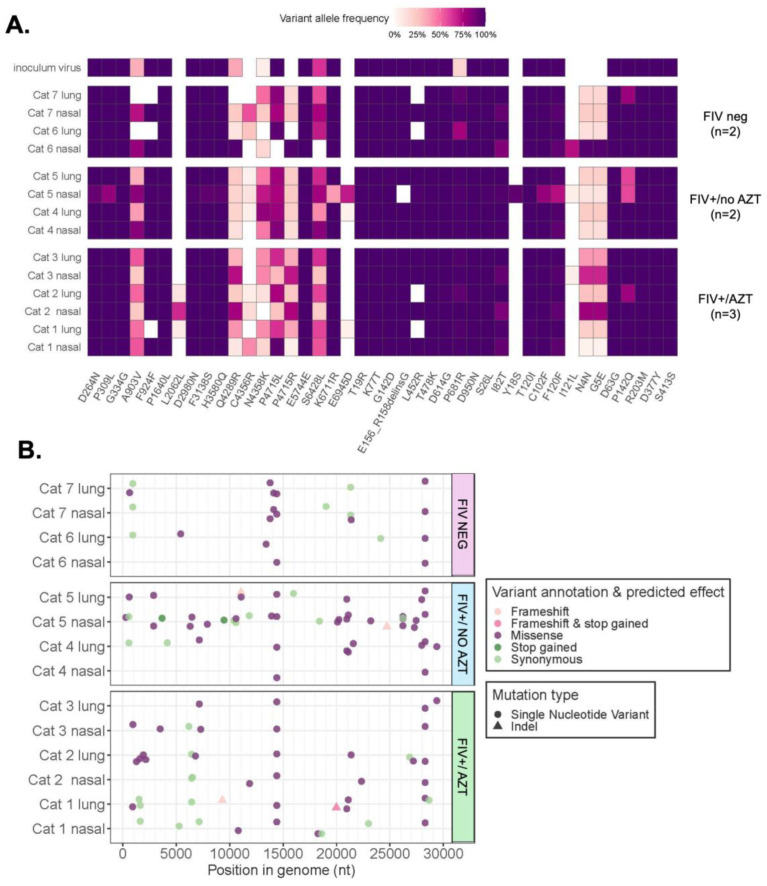
(**A**) A variant map across the SARS-CoV-2 genome encompasses all mutations detected at greater than 3% allele frequency within the viral inoculum, as well as in RNA recovered from cat lung and nasal turbinate tissues. Each row represents a sample, and each tile represents a mutation, shaded by the allele frequency at which it was observed. Code for visualizing this plot was adapted from the R package ‘outbreak.info, version 4.3.3’. (**B**) Seventy-six SARS-CoV-2 variants were detected in cats but not in the viral inoculum. Cat identifier and tissue type of each sample are indicated on the left, and treatment group is indicated on the right of the y-axis. Variants are cataloged by type, predicted effect, and nucleotide (nt) position in the viral genome on the x-axis.

A map of SARS-CoV-2 genomic variation was visualized by aligning all the sequences generated from both the cat and the inoculum RNA to a wildtype reference sequence, MN908947.3, which is derived from the Wuhan-Hu-1 isolate (Figure 7A,B). In the seventy-six variants observed in viral RNA recovered from cats but not detected at any level in the viral inoculum, a predominance of missense or nonsynonymous single-nucleotide variants was noted (Figure 7B). The bioinformatic analysis revealed the emergence of new mutations and the absence of other mutations in viral RNA recovered from cats (Table 2). Furthermore, certain mutations present in the viral inoculum appeared to be amplified in cats, whereas others appeared to be weakened. In the spike gene, the L452R mutation was present at a high allele frequency in the inoculum while it was absent in lung tissue in both cat groups: FIV+/AZT and FIV-negative. In addition, the P681R mutation was presented at less than a 25% allele frequency in the inoculum; meanwhile, it was significantly present in cat viral RNA (Table 2).

## 4. Discussion

This study establishes the feasibility and potential for domestic cats to contribute as a translational model to study the effects and treatment of COVID-19 in people with HIV. Additionally, this study highlights the possibility that infections with immune deficient viruses followed by SARS-CoV-2 coinfection affect histopathologic changes and the persistence of detectable SARS-CoV-2 viral RNA in samples collected from infected animals. Viral mutations detected after persistent passage through immune-deficient animals or people may contribute to virulence, alterations in transmission, host specificity, and even immune evasion. These concerns underscore the need for an animal model to better study the effects of SARS-CoV-2 infections in animals or people with lentiviral infections.

This study established an FIV infection model that resulted in detectable FIV viral load in plasma, similarly to what is expected with the asymptomatic phases of natural FIV infection in cats [52,53,54]. After SARS-CoV-2 infection, FIV viral RNA load in plasma significantly decreased while the acute SARS-CoV-2 infection occurred. The diminished FIV load may be explained by the immune stimulation and increase in circulating T lymphocyte populations in response to the coinfection. This decrease in FIV viral RNA was noted regardless of AZT treatment. Within this study, SARS-CoV-2 viral RNA was detected in the plasma of all cats at 3 days post-SARS-CoV-2 inoculation but was no longer detected in the plasma by 5 days post-inoculation in all but one cat. This cat with persistent SARS-CoV-2 viremia not only had significantly increasing plasma viral loads through day 5 but was also one of the two cats that was FIV-infected and untreated for that FIV infection. The limited sample size prevents regarding this finding as significant, but this finding suggests that the role of untreated lentiviral infection in persistent COVID-19 should be explored further. 

While limited in sample size and scope, this study offers insight into the complexity of the model. Research in people with HIV who are coinfected with SARS-CoV-2 often provides conflicting results. On the one hand, some studies conclude that people living with HIV who are subsequently infected with COVID-19 had no increased risk or severity of SARS-CoV-2 infection compared with people who do not live with HIV; however, most of these studies encompass a population of people living with HIV who have access to treatments and resources for their infection [55,56]. In contrast, other studies conclude that people living with HIV and low CD4^+^ T lymphocyte levels who are coinfected with SARS-CoV-2 have worsened outcomes and require more frequent hospitalization [57]. In addition, depressed CD4+ T lymphocyte responses infer a lack of access to treatments or resources needed to improve these cellular levels and functions. In our study, the plasma viral load of SARS-CoV-2 was not only persistent but increased on day 5 post-SARS-CoV-2 inoculation in one FIV+/no AZT cat. Not surprisingly, the untreated FIV+ cats tended toward lower percentages of CD4+ T lymphocytes as compared to the FIV+/AZT cats. An expanded study with a larger sample size is needed to support the significance of the administration of a proper antiretroviral therapy for these immunocompromised patients in order to improve immune recovery and avoid worsened clinical outcomes with coinfection [28]. An expanded study would also allow for the inclusion of an AZT-treated FIV-negative group to better delineate the effects of AZT treatment alone on immune response and viral replication for specific variants. This domestic cat model shows great potential in investigating these clinically applicable research questions. Additionally, this model can be used to investigate parameters of successful, novel therapeutic approaches, but these parameters will need to be evaluated beyond plasma viral loads since SARS-CoV-2 is undetected in most cats by 5 days post-inoculation. Persistent infection in other, localized areas must be more thoroughly evaluated in conjunction with any subacute or chronic histopathologic changes.

The immunophenotype changes were investigated by measuring CD4+ and CD8+ T lymphocyte numbers in plasma before and after SARS-CoV-2 inoculation. It is well established that CD4+ cell counts are critical drivers of coinfection vulnerability and worsened outcomes in people living with HIV. Data collected from hospitalized HIV-infected people who were coinfected with SARS-CoV-2 concluded that a reverse relationship exists between CD4+ cell counts and mortality rate [9,58]. In addition, Coleman et al. and Frieman et al. established that individuals lacking the administration of proper antiretroviral therapy experience lower CD4+ T lymphocyte counts and a subsequent failure to suppress viral coinfection load [9]. In our study, FIV+ cats that were treated with AZT and the FIV-negative cats both had improved percentages of CD4+ T lymphocytes through 5 DPI SARS-CoV-2 infection when compared to the untreated FIV+ cats. This result supports that usage of a proper antiretroviral therapy works to increase percentage of CD4+ T lymphocytes in these otherwise immune-deficient patients and offers some protection from severity of SARS-CoV-2 coinfections. The administration of AZT did not alter the percentage of CD8+ T lymphocytes in this study between groups, with no significant group differences noted post SARS-CoV-2 inoculation in both FIV+/AZT and FIV+/no AZT cats. To better investigate these findings, there is need for expanded studies to thoroughly examine these immunophenotypic alterations with coinfections that involve lentiviral immune deficiency as frequently seen with HIV and FIV.

While none of the cats in this study developed marked clinical disease after SARS-CoV-2 infection, serious disease is often not seen in people or animals infected with SARS-CoV-2. Several studies also conclude limited to no increases in clinical severity with COVID-19 in people living with HIV, especially with normal CD4+ T lymphocyte counts due to effectively administered antiretroviral therapy [59,60,61,62]. Moreover, a study in 2022 reported asymptomatic SARS-CoV-2 among HIV patients in numbers similar to the general population [63]. Despite limited differences in clinical disease, histopathologic analysis showed significant respiratory disease in both upper and lower airways, and acute lung inflammation is noted in all SARS-CoV-2-infected cats with several similarities to that seen in people with COVID-19. A more thorough analysis with a larger sample size is needed to make significant conclusions; however, in this particular study, untreated FIV+ cats had significantly more perivascular inflammation than other groups, and the FIV-negative cats had significantly less pulmonary edema than cats infected with FIV. In contrast, AZT-treated FIV+ cats had significantly more serous exudate and pulmonary edema than other groups. Several histopathologic features mimic those noted in previous publications with lesions in infected cats similar to that seen in people with COVID-19 [46]. SARS-CoV-2 viral loads in collected tissues were significantly increased in nasal turbinate and tracheal samples as compared to lymph nodes or lower respiratory tract samples, regardless of FIV infection status or treatment category. The preference for upper respiratory targets may contribute to viral transmission through nasal shedding. Decreased SARS-CoV-2 viral loads were noted in lymph nodes from the FIV+/AZT group, suggesting some restoration of the immune response due to the application of antiretroviral therapy, especially since one of the mechanisms of action of Zidovudine is to improve immunological status [35,64].

One of the most valuable outcomes from this study is the sequencing of SARS-CoV-2 genomes isolated from infected lung tissues and nasal turbinate tissues from the three treatment groups. An analysis of SARS-CoV-2 viral RNA recovered from infected cats unveiled a spectrum of within-host evolutionary events from viral inoculum to passage through a natural host, the domestic cat. These changes include emergent mutations, the absence of certain established mutations, and alterations in mutation frequency distributions. For instance, the L452R mutation, recognized for its pivotal role in Delta Variant’s heightened viral infectivity and attributed to augmented viral binding affinity towards angiotensin-converting enzyme (ACE) with intensified viral replication, was notably absent in the lung tissue of cats from the FIV-negative groups and those concurrently FIV+/AZT (*n* = four out of five cats) [65,66,67,68]. This may be related to the influence of host species on viral genomic adaptation. The prominence of missense (nonsynonymous) single-nucleotide variants (SNVs) assumes particular significance, as these changes induce amino acid changes. Nonsynonymous variants may denote selective pressures at specific loci for bolstered binding and ingress into feline host cells, evasion of host immunity, and other crucial species-specific functionalities. Positive selection for nonsynonymous mutations was evident at the virus population level, in the ORF1ab gene, and from collected lung samples. Virus genomes recovered from FIV+ cats not given AZT also showed signs of positive selection for nonsynonymous mutations. The numerous mutations and their relative importance highlight the concerns regarding viral evolution after passage through both animal hosts as well as through immunodeficient hosts. This proposed feline model holds tremendous potential for further evaluating these phenomena.

In summary, this study investigated the potential for the use of FIV-infected domestic cats as a translational model to study the coinfection of SARS-CoV-2 in people living with HIV. Our findings support this model for the study of pathogenesis, prevention, and therapeutic interventions that offer hope to people with COVID-19 who may be immune-suppressed due to concurrent HIV infection. Our studies support the positive effect of the administration of proper antiretroviral therapy to protect from disease severity in COVID-19 and reduce viral shedding, but the limitations in animal numbers between groups limit the detection of significance and warrant further exploration. The reduction in infection length and viral loads will inevitably reduce the rates of viral mutations, which may result in variations of SARS-CoV-2 that can have increased virulence, increased disease, enhanced transmission, or even immune evasion [17,18]. While this pilot study shows promise, significant limitations exist in this study due to the poor sample sizes of the groups; however, trends noted in this small study support the need for a follow-up investigation with a larger cohort. Further studies are needed to clarify the evolutionary pressures exerted on SARS-CoV-2 during the infection of immunodeficient hosts. Future studies can also assess the transmission of these viral infections and interventions that may be necessary to reduce negative outcomes in these patients.

## Figures and Tables

**Table 1:** Clinical parameter scoring system for feline respiratory disease. The provided table outlines a modified version of a previously utilized clinical scoring system (see text).

		Clinical Score			
		0 (Healthy)	1 (Mild)	2 (Moderate)	3 (Marked)
**Assessed/Observed Clinical Parameter**	**Body Weight**	No weight loss	<5% weight loss	5 to 10% weight loss	>10% weight loss
	**Temperature**	37.2 to 39.0 °C	39.1 to 39.4 °C	39.5 to 39.7 °C	>39.7 °C
	**Pulse Oximetry**	98 to 100%	96 to 97%	93 to 95%	<93%
	**Activity**	Normal	Mild reduction when disturbed * (mild lethargy)	Moderate reduction when disturbed * (moderate lethargy)	Little to no activity disturbed * and reduced activity when stimulated **
	**Behavior**	Normal	Noticeable but minimal reduction in interest in food and/or attention	Moderate reduction in interest in food and/or attention	Anorexia and/or complete lack of interest
	**Respiratory** **Effort**	Normal resting respiratory effort and rate	Mild tachypnea (>35 breaths per minute at rest) with no overt increase in respiratory effort	Moderate tachypnea (>40 breaths per minute at rest) with moderate increase in effort	Marked tachypnea (>45 breaths per minute at rest) with marked dyspnea or effort
	**Ocular and/or Nasal** **Discharge**	None	Mild discharge observed from nares or eyes	Moderate discharge observed from either nares or eyes or from both nares and eyes	Marked or purulent discharge noted from nares and/or eyes
	**Coughing**	None	Occasional, rare cough observed	Intermittent coughing (at least one episode per 30 min)	Marked, persistent coughing (2+ episodes per 30 min)
	**Wheezing**	None	Occasional, rare wheeze observed	Intermittent wheezing (at least one episode per 30 min)	Marked, persistent wheezing (2+ episodes per 30 min)

* Disturbed: observer in the room but kennel unopened. ** Stimulated: kennel opened.

**Table 2:** This table summarizes the specific mutation sites with changes between inoculum and samples collected from infected cats as presented in the variant map. “**Emergent**” refers to instances of mutations at a genetic locus that were not evident in the inoculum but were evident in at least one cat from a specific treatment group. “**Absence**” refers to mutations at a genetic locus that were evident in the inoculum but were not evident in at least one cat from a specific treatment group. “**Amplified**” indicates mutations with a % increase in allele frequency between the inoculum at least one cat in a treatment group. “**Weakened**” indicates mutations with a % decrease in allele frequency between the inoculum and at least one cat in a treatment group. Each ●/◊/Δ represents a single cat from one of three treatment groups, as noted in the table legend. Gene products affected by that specific site are listed in the far-right column. ●: FIV+/AZT (*n* = 3). ◊: FIV+/No AZT (*n* = 2). Δ: FIV-negative (*n* = 2).

Mutation	Emergent (Absent → Present)	Absence (Present → Absent)	Amplified (Present → More Present)	Weakened (Present → Less Present)	Protein
**C4356R**	●● ◊◊ ΔΔ				ORF1ab
**P4715R**	●●● ◊◊ ΔΔ				
**E6945D**	● ◊◊				
**A903V**		ΔΔ			
**L2062L**	●●				
**F924F**		● ΔΔ			
**N4358K**			◊◊		
**L452R**		●● ΔΔ			Spike
**P681R**			●●● ◊◊ ΔΔ		
**E156**		◊			
**N4N**	●●● ◊◊ ΔΔ				N
**G5E**	●●● ◊◊ ΔΔ				
**P142Q**				●	
**Y18S**	◊				ORF7
**I121L**	● Δ				ORF8

## Data Availability

The data supporting the findings of this study are openly available in the Zenodo repository at https://zenodo.org/records/11402714 (accessed on 11 April 2024); https://doi.org/10.5281/zenodo.11402714, or contained within the provided figures and Supplementary Table S1.

## Supplementary Materials

The following supporting information can be downloaded at: https://www.mdpi.com/article/10.3390/microorganisms12071289/s1, Table S1: Baseline characteristic data for animal groups.
