# Supplementary material for: Utilizing Feline Lentiviral Infection to Establish a Translational Model for COVID-19 in People with Human Immunodeficiency Virus Infection
Source: Microorganisms. 2024 Jun 25;12(7):1289. doi: 10.3390/microorganisms12071289 (PMC11278576; doi:10.3390/microorganisms12071289)
Supplement: Supplementary file 1 [file microorganisms-12-01289-s001.zip › microorganisms-3007012-supplementary.pdf]

microorganisms

MDPI

Table S1. Baseline characteristic data for animal groups. This table outlines the cohort populations at the time of AZT treatment of vehicle therapy initiation.

Animal ID	Sex	FIV Status	Treatment Group	Age (Years)	Body Weight (kg)
6902	F	+	AZT	1.8	3.86
3663	F	+	AZT	1.7	3.67
7982	F	+	AZT	1.8	4.52
7066	M	+	Vehicle	1.8	4.09
6297	F	+	Vehicle	1.8	3.98
3054	M	-	Vehicle	0.9	5.75
7079	M	-	Vehicle	2.0	3.71
